# Functional Outcome of Surgical Stabilisation of Acetabular Fractures

**DOI:** 10.5704/MOJ.2107.019

**Published:** 2021-07

**Authors:** NH Fakru, WI Faisham, D Hadizie, S Yahaya

**Affiliations:** Department of Orthopaedics, Universiti Sains Malaysia, Kubang Kerian, Malaysia

**Keywords:** acetabular fracture, functional outcome, Matta radiological outcome

## Abstract

**Introduction:**

Approach to the management of displaced acetabular fractures has evolved from conservative to operative management after the work of Judet and Letournel. Various surgical methods have been explored and described by authors to address this type of fracture, leading to improved clinical outcome. This study aimed to evaluate functional outcome of surgically treated displaced acetabular fractures in the Malaysian context.

**Materials and methods:**

We analysed 43 patients with isolated acetabular fractures who were treated operatively with a minimum of three years follow-up. Anthropometric data, Judet-Letournel fracture pattern, surgical approach and complications were recorded. Post-operative Matta radiological outcome were evaluated for joint congruency and hip functional outcome was evaluated using Merle d’Aubgine-Postel and Harris Hip Score (HHS). All statistical analyses were analysed using SPSS version 24.0.

**Results:**

The most frequent elementary fracture type was posterior wall (30.2%) while associated type was both columns (23.3%). Mean functional outcome of Merle d'Aubigné-Postel was 15.77 and HHS was 86.6. Thirty-three (76.7%) patients achieved satisfactory functional outcome, 19 (44.1%) patients achieved anatomic reduction (<2 mm step-off) based on Matta classification while 24 (55.8%) did not achieve the desired outcome. Fracture pattern exhibited strong association with post-operative Matta radiological outcome (p-value 0.001). However both fracture pattern and Matta radiological outcome did not exhibit association with the functional outcome group. The mean time for surgical interventions was 10.8 days and there was no significant association with final functional outcome score.

**Conclusion:**

Fracture pattern is a strong contributing factor towards post-operative Matta radiological outcome. However, achieving the perfect anatomical reduction is not of utmost important factor to predict the good functional outcome.

## Introduction

The incidence of acetabular and pelvic fractures in the United Kingdom is approximately three per 100 000 per year, which constitutes 2% to 8% of all fractures^1,2^. However, data for the prevalence of the acetabular fractures in South East Asia, particularly in Malaysia are scarce. These types of fractures are usually the results of high-energy trauma i.e., road traffic accidents or falls from height, which are often associated with other life-threatening injuries such as intracranial bleed, intrabdominal and lung injuries^3^.

The surgical management of acetabular fractures poses paramount challenges to the trauma surgeons due to the complexity of the anatomy and surgical approaches. Usual complications resulted from the approaches such as, 20% to 25% of the patients are with poor functional outcomes in the medium-term follow-up such as concentric reduction of hip, articular surface congruency, and loss of femoral head blood supply as the ultimate outcome, including degenerative changes in the hip joint^3,4^. However, if the fractures are left unreduced, patients with displaced acetabular fractures are at risk of developing early hip osteoarthritis^5^.

Therefore, operative management has become the standard approach adopted by most surgeons worldwide. If operative management is to be considered, the surgical approach and accuracy of the reduction are strongly influenced by the surgeon’s training and expertise. The number of successful anatomic reductions is also directly related to the number of operations performed by the surgeon himself^6^.

Hence the aim of this study is to evaluate the functional outcome of surgically treated displaced acetabular fracture by using both modified Merle d’Aubgine and Harris Hip Scores, and to evaluate radiological outcome by using the Matta classification according to Judet-Letournel type of fracture.

## Materials and Methods

We conducted a retrospective cross-sectional study to evaluate the functional outcome of surgical stabilisation of acetabular fractures. Subjects were patients admitted with acetabulum fracture between the year 2008 to 2014 who underwent surgical treatment. However, patients with head and spinal injuries with neurological deficit, ipsilateral femoral head, femoral neck, complex femur and tibia fractures were excluded from this study. The detailed epidemiological data and injuries were then extracted and evaluated from the patients’ folders. Fracture characterisation was evaluated by PAC radiology system using standard preoperative digital radiographs of antero-posterior pelvis and Judet’s obturator and iliac oblique views. Final confirmation of fracture pattern was done using computed tomography (CT) with 3-D reconstruction and assessed by single assessor and a radiologist.

The surgery for posterior wall and columns was done within three days of the injury and the anterior approach for bicolumnar and anterior wall was done after five days of the trauma to minimize the risk of bleeding. The posterior wall and column fractures were explored through the Kocher-Langenbeck approach and stabilised using reconstruction plate and screwed to the posterior wall spring plate in selected cases. The transverse fractures or the both associated columns fractures were explored through modified Letournel approach and stabilised using curve reconstruction plate over the pelvic brim and quadrilateral plate in selected cases.

Post-operative rehabilitation was initiated on day three with passive and active abduction using skateboard exercise. Patients were prescribed with strict non-weight bearing exercises for six weeks using crutches and were taught active flexion and abduction exercises. The patients then had follow-up radiographs at six weeks, before being allowed for weight bearing. Weight bearing started in a gradual manner, from toe touch to 10 kg of weight to full weight bearing at 12 weeks. The patients were reviewed every three months clinically and radiographically for symptoms like hip pain, severe reduced hip ROM and also limping, and early complications like infections, severe hips arthrofibrosis, limb length discrepancy, hip arthritis, and avascular necrosis.

The post-operative radiograph was evaluated based on Matta Classification; anatomic (< 2mm fracture gap or step off deformity), imperfect (2-3mm gap or step off) and poor (more than 3mm gap or step off). Those who had imperfect and poor reduction were categorised as non-anatomic. The functional outcome was assessed in the clinic at least two years after the surgery by a single assessor based on modified Merle d’Aubginine and Harris Hip Score via interviews and physical examinations.

This study was approved by the Research Ethics Committee (Human) Universiti Sains Malaysia [USMKK/PPP/JEPeM (264.4(1.4)]. All data were analysed using SPSS (version 24) statistical software.

## Results

A total of 43 acetabular fractures were evaluated from 41 adults and two adolescents inclusive of 29 (67.4%) males and 14 (32.6%) females. Mean age of the patient was 36.14 (± 13.43) ranging from 18 to 61 years old at the time of evaluation. The reasons of injury were largely contributed by motor vehicle accidents (95.3%) while only two patients (4.7%) were injured due to fall from a significant height. All patients underwent surgical intervention for at least two years prior to evaluation (ranged 2 -12 years). The surgeries for all samples were performed by three experienced surgeons.

As for the fracture patterns cases, 18 (41.9%) were elementary and 23 (53.6%) associated. Among the patients, two (4.7%) presented with acetabular physeal injuries during the age of 13 and 14 respectively. The most frequent elementary fracture pattern was posterior wall (13 cases, 30.2%) while the most frequent associated type was both columns fractures (10 cases, 23.3%), followed by transverse + posterior wall (8 cases, 18.6%) (Table I).

**Table I:** The proportion of fracture type and its functional outcome based on modified Merle d’Aubigné-Postel score and the Harris Hip score

Fracture type	n	(%)	Merle d’Aubigné score		The Harris Hip score	
			Satisfactory	Unsatisfactory	Satisfactory	Unsatisfactory
			n (%)	n (%)	n (%)	n (%)
Elementary						
Posterior wall	13	(30.2)	10 (76.9)	3 (23.1)	10 (76.9)	3 (23.1)
Posterior column	2	(4.7)	2 (100.0)	0 (0)	2 (100.0)	0 (0)
Anterior wall	-	-	-	-	-	-
Anterior column	1	(2.3)	1 (100.0)	0 (0)	1 (100.0)	0 (0)
Transverse	2	(4.7)	1 (50.0)	1 (50.0)	1 (50.0)	1 (50.0)
Associated						
T-shaped	2	(4.7)	1 (50.0)	1 (50.0)	1 (50.0)	1 (50.0)
Post. wall + post. column	2	(4.7)	1 (50.0)	1 (50.0)	1 (50.0)	1 (50.0)
Transverse + post. wall	8	(18.6)	7 (87.5)	1 (12.5)	7 (87.5)	1 (12.5)
Ant. Column + post.hemitransverse	1	(2.3)	1 (100.0)	0 (0)	1 (100.0)	0 (0)
Both columns	10	(23.3)	7 (70.0)	3 (30.0)	8 (80.0)	2 (20.0)
Physeal	2	(4.7)	2 (100.0)	0 (0)	2 (100.0)	0 (0)

The mean functional outcome of modified Merle d’Aubigne was 15.77 and Harris Hip Score (HHS) was 86.6. Thirty-three (76.7%) patients achieved satisfactory functional outcome, 11 patients (25.6%) were graded as excellent, 22 (51.2%) as good, five (11.6%) as fair, and five (11.6%) as poor. Nineteen (44.1%) patients achieved anatomic reduction while 24 (55.8%) resulted otherwise. Among 24 non-anatomic group, five (11.6%) patients graded as poor and 19 (44.2%) as imperfect (Table I). Both fracture pattern and Matta radiological outcome did not exhibit association with functional outcome group (Table II). However, strong association was found between fracture pattern and Matta radiological outcome (p value 0.003) (Table III). The mean time for surgical interventions was 10.8 days (ranged 2-21 days) and there was no significant association with the final functional outcome score. There was no statistical difference of functional outcome assessed by both scoring systems.

**Table II:** Association between acetabular fracture pattern and Matta radiological outcome with functional outcome Modified Merle d'Aubigné-Postel and Harris Hip score

		Modified Merle d'Aubigné			Harris Hip Score		
		Satisfactory	Unsatisfactory	p-value^a^	Satisfactory	Unsatisfactory	p-value^a^
Fracture	Elementary	14 (77.80	4 (22.2)	>0.05	14 (77.8)	4 (22.2)	>0.05
	Associated	17 (73.9)	6 (26.1)		18 (78.3)	5 (21.7)	
Matta reduction	Anatomic	15 (45.5)	4 (40.0)	>0.05 (1.00)	15 (44.1)	4 (44.4)	>0.05 (1.00)
assessment	Non-anatomic	18 (54.5)	6 (60.0)		19 (55.9)	5 (55.6)	

^a^Fisher exact test

**Table III:** Association between acetabular fracture pattern and anatomic/non-anatomic Matta Radiological outcome (n= 41, exclude 2 physeal injuries)

Fracture type	Matta Radiological outcome		X^2^ stat (df)	p-value^b^
	Anatomic n (%)	Non-anatomic n (%)		
Elementary	13 (72.2)	5 (27.8)	8.64 (1)	0.003
Associated	6 (26.1)	17 (73.9)		

^b^Chi-square statistics

As for the surgical aspect, 13 (30.2%) patients had surgical complications in which four had surgical site/implant related infections followed by septic arthritis and osteomyelitis of hip, which required multiple debridement and ultimately removal of implants. Another three patients required total hip replacements while one was under oral antibiotics and close observation at the time of the evaluation. Other recorded complications were post-operative heterotrophic ossification, joint stiffness, joint pain, mild to moderate osteoarthritis and veno-thromboembolism.

## Discussion

Surgical stabilisation of a displaced acetabular fracture is currently the treatment of choice because it allows anatomical reconstruction of the hip joint^1,2,7-9^. The goal of surgical treatment is to provide good hip function which is pain free and to achieve full range of motion (ROM). Early definitive stabilisation of acetabular fractures is necessary as it facilitates early rehabilitation and restores the normal hip function^5,10-13^. However, this is not always feasible since these patients often have various physiological insults that require stabilisation prior to treating the fracture^7^.

Our study found that posterior wall fracture was the commonest type of fracture pattern, accounting to 30.2% followed by both column fractures, 23.3% and transverse with posterior wall fracture, 18.6%. There was no anterior wall fracture encountered in this study and the rest of fracture types were rare, ranging from 2.3% to 4.7%. Laird *et al* and Giannoudis *et al* in their large series of studies also reported similar distribution of the commonest to the least fracture pattern to occur^1,3^. However a local study conducted by Anizar-Faizi *et al* reported that almost half (46.7%) were of posterior wall type, 20% were both columns and 6.7% were transverse—transverse with posterior wall, anterior column with posterior hemitransverse and posterior wall + posterior column, respectively^11^.

It was also found that the severity of fracture played an important role in achieving a perfect anatomical reduction intra-operatively as such fracture pattern exhibited strong association with post-operative Matta radiological outcome (p value 0.003). Majority of elementary fracture pattern (72.2%) achieved anatomical reduction on initial radiographs, while majority of associated group (73.9%) resulted otherwise. This indicated that the more complex fracture pattern, the poorer the scores obtained from Matta Classification.

This study also found that, 77.8% of the elementary fracture type had satisfactory functional outcome by modified Merle d'Aubigné and HHS, 76.9% of posterior wall fractures, 100% of both anterior and posterior column fractures and 50 % of transverse fractures had good to excellent outcome. Whereby among the associated group, 70% both columns fracture, 87.5% transverse + posterior wall fracture, and 100% anterior column + posterior hemitransverse fracture exhibited good to excellent outcome. Statistically, our study revealed that there was no association between fracture pattern and functional outcome (p value 1.00). Giannoudis et el found similar finding in which patients with associated fracture types and those with injuries to the anterior wall and posterior column were the most likely to have a poor functional outcome^3^ and they were further supported by Mears *et al*^14^. Another similar finding, Matta *et al* noted that T-shaped and posterior wall fractures were associated with a poor functional outcome^15^ and the findings were further supported by Murphy *et al* that functional outcome was related to associated fracture types^13^.

**Fig. 1:**
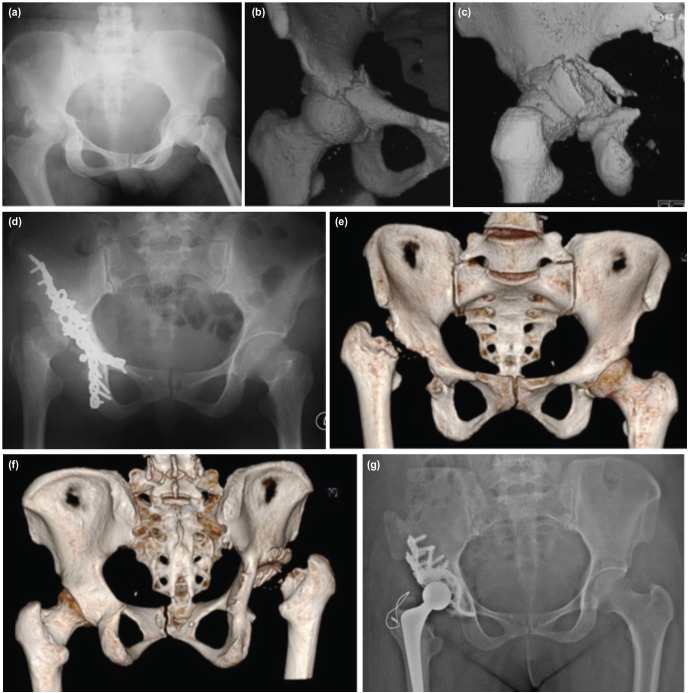
Transverse bicolumnar fracture with posterior column injury in 54-year-old lady was stabilised with double approach. It was complicated with infection requiring removal of all implants and prolonged antibiotic treatment. (a) Anteroposterior view radiograph shows a displaced transverse bicolumnar fracture with posterior column injury. (b) Closed up anteroposterior CT scan image of the fracture. The fracture line extends from the anterior column, traversing to the posterior. (c) Closed up posterior view of CT image shows comminuted medial and posterior wall. (d) Anteroposterior pelvic radiograph of bicolumnar plating of the acetabulum. This patient succumbed to implant related infection which lead to septic arthritis and AVN with femoral head lysis. (e) Anteroposterior view of CT image of right hip after removal of implant and remaining collapsed femoral head. Note part of anterior wall has undergone lysis too. (f) CT image posterior view of right hip shows the absent posterior wall of acetabulum. (g) There was no femoral head to build up the posterior wall, the illiac crest was used to build up posterior wall with impacted graft and cage cemented arthroplasty.

In addition, the quality of surgical reduction is crucial in the management of acetabulum fracture. Poor quality of reduction in the weight-bearing dome of acetabulum carries a poor prognosis^6,16-18^ but anatomical reduction does not always result in a good outcome^2,16^. In this study, 54-56% patients with non- anatomic reduction achieved satisfactory functional outcome (good and excellent) when used both modified Merle d'Aubigné and HHS. Fifteen of them (34.8%) had imperfect reduction (fracture gap / articular step off 2-3mm), while the other three (7%) poor reduction (fracture gap / articular step off >3mm). Three out of seven patients who scored poor functional outcome both by Merle d'Aubigné and HHS had anatomic reduction on initial radiographs, and one had imperfect reduction. They acquired implant related infection post-operatively and underwent multiple debridement of the previous surgical sites, multiple hospital admissions with prolonged antibiotics and ultimately development of hip avascular necrosis. Three of them had total removal of the implants while another one had two screws removal from the hip. Despite of the initial non-anatomic reduction (Fig. 1), post-operative remodelling of the fracture with minimal or absent arthritic changes were seen in the serial follow-up radiographs after several years, and might attribute to the good and excellent scores in those patients.

**Fig. 2:**
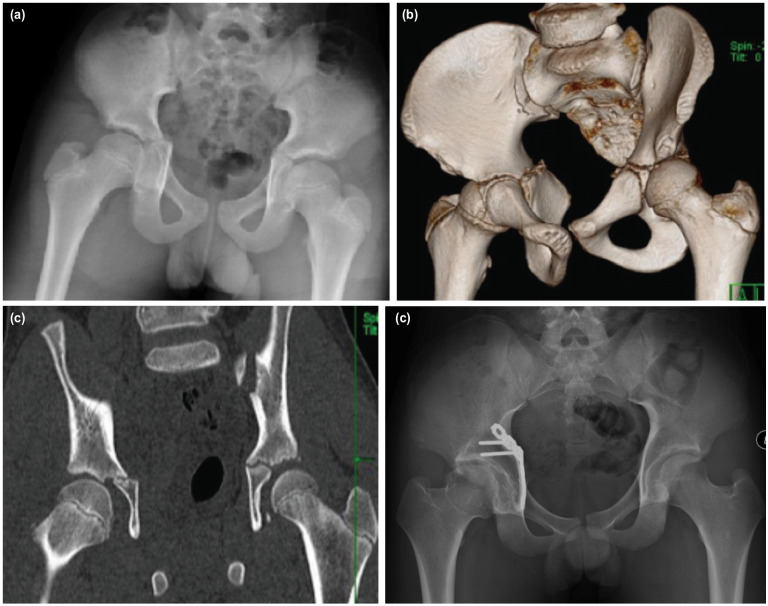
A 13-year-old boy pillion rider sustained a displaced trans-physeal fracture over right acetabulum. (a) Initial pelvis AP radiographs shows a displaced transphyseal acetabular fracture. (b) 3D CT image of pelvis depicted the acetabular physeal involvement of fracture. (c) AP image of CT scan. (d) Stabilisation was done using buttress quadrilateral plate with screws avoiding the acetabular physis. (e) There is evidence of femoral head avascular necrosis and subsequently well-remodelled six years after injury (e). Both modified Merle d'Aubigné-Postel and HHS functional outcome are excellent.

As for the rate of infection in our study, it was 9.3% (four patients) and this number was slightly higher compared to other studies^3,11,19-21^. This condition was observed in those who needed double surgical approaches with longer surgical time. Initially they developed surgical site infection which later progressed into deep-seated infection and two of them succumbed to complication of septic arthritis and chronic osteomyelitis. Due to that, debridement was performed as well as the removal of implant in addition to intravenous and oral antibiotics for a minimum of six weeks. As for the follow-up, hip arthritis and avascular necrosis (AVN) were observed attentively. Among the four patients, one had total replacement surgery (Fig. 1) while the other three refused surgery. Due to the delay in rehabilitation and spread of the infection, the functional score in these patients was unsatisfactory (fair to poor). Hip AVN was also seen in one of the acetabular physeal injury patient, secondary to undiagnosed neck of the femur fracture which was not picked up on initial radiographs and CT scan. Fortunately, he scored a satisfactory functional outcome in both modified Merle d'Aubigné and HHS and most likely attributed to good remodelling of fracture after several years of post fixation (Fig. 2). All patients in our study received post-operative oral celecoxib 200mg for two weeks as analgesia control and heterotrophic ossificans prevention. Despite of the medications, two patients had HO and one had DVT which required IVC filter.

Our study is an improvement on past studies, as we used both modified Merle d'Aubigné-Postel and Harris Hip Score as the tools to evaluate the functional outcome while most authors only used single scoring system. However, this study experienced some limitations, for instance, this study was conducted in a single centre with relatively small number of participants, whereas multicentre involvement with much larger sample size is preferred in producing better statistical analysis. Another limitation is on the issue of compliance to post-operative rehabilitation that was not adequately addressed in this study. Further, strict adherence to the rehabilitation program was one of the main contributing factors to good clinical outcome.

## Conclusion

Fracture pattern has strong contribution factor towards postoperative Matta radiological outcome as the severity of the fracture can influence the quality of initial reduction. However, achieving the perfect anatomical reduction is not the utmost important factor when fixing the fracture as imperfect reduction also yields equally good to excellent functional outcome. Controlling the infection, minimising the intra-operative and post-operative complications and strict adherence to the post-operative rehabilitation program contribute to better prognosis in treating the acetabular fracture surgically.
